# Neuroinflammatory Mechanisms in Ischemic Stroke: Focus on Cardioembolic Stroke, Background, and Therapeutic Approaches

**DOI:** 10.3390/ijms21186454

**Published:** 2020-09-04

**Authors:** Carlo Domenico Maida, Rosario Luca Norrito, Mario Daidone, Antonino Tuttolomondo, Antonio Pinto

**Affiliations:** 1 U.O.C di Medicina Interna con Stroke Care, Dipartimento di Promozione della Salute, Materno-Infantile, di Medicina Interna e Specialistica di Eccellenza “G. D’Alessandro”, University of Palermo, 90127 Palermo, Italy; rosario94.norrito@gmail.com (R.L.N.); mario.daidone@unipa.it (M.D.); antonino.tuttolomondo@unipa.it (A.T.); carlodomenico.maida@icloud.com (A.P.); 2 Molecular and Clinical Medicine PhD Programme, University of Palermo, 90127 Palermo, Italy

**Keywords:** ischemic stroke, cardiac embolism, neuroinflammation

## Abstract

One of the most important causes of neurological morbidity and mortality in the world is ischemic stroke. It can be a result of multiple events such as embolism with a cardiac origin, occlusion of small vessels in the brain, and atherosclerosis affecting the cerebral circulation. Increasing evidence shows the intricate function played by the immune system in the pathophysiological variations that take place after cerebral ischemic injury. Following the ischemic cerebral harm, we can observe consequent neuroinflammation that causes additional damage provoking the death of the cells; on the other hand, it also plays a beneficial role in stimulating remedial action. Immune mediators are the origin of signals with a proinflammatory position that can boost the cells in the brain and promote the penetration of numerous inflammatory cytotypes (various subtypes of T cells, monocytes/macrophages, neutrophils, and different inflammatory cells) within the area affected by ischemia; this process is responsible for further ischemic damage of the brain. This inflammatory process seems to involve both the cerebral tissue and the whole organism in cardioembolic stroke, the stroke subtype that is associated with more severe brain damage and a consequent worse outcome (more disability, higher mortality). In this review, the authors want to present an overview of the present learning of the mechanisms of inflammation that takes place in the cerebral tissue and the role of the immune system involved in ischemic stroke, focusing on cardioembolic stroke and its potential treatment strategies.

## 1. Introduction

The appearance of focal neurological signs or symptoms with a vascular cause that endures at least 24 h characterizes stroke; brain MRI or CT in baseline conditions and brain CT with contrast medium after 48–72 h must verify the presence of ischemic lesions. Stroke is the third highest cause of death and the most common motive of invalidity in the occidental part of the world. It affects 15 million people per year in the world, 5 million of them die, and the other 5 million are permanently physically challenged [1]. The incidence of stroke is correlated with ethnicity; for example, in the United States, the hazard of pathology is increased in Black and Hispanic people than in Caucasians as reported by multiple studies [1]. Stroke incidence in men is about 62.8 per 100,000 while women have a stroke incidence of 59 per 100,000; this suggests that this disease affects more men than women. However, this detection concerns only young subjects; in fact, the American Heart Association recently suggested that women aged more than 75 are more affected than men of the same age [2]. Furthermore, the chance of stroke is proportional to the age, and nearly 75% of all strokes take place in patients older than 64 years [1] even though one-third of the cases of strokes occurs in younger persons proposing that this pathology does not affect just older people. Regarding the pathophysiology, we can identify a couple of principal categories of stroke utterly different from each other, hemorrhage and ischemia. Hemorrhage is represented by the presence of blood in the cerebral parenchyma able to accumulate and press on the adjacent parenchyma. In contrast, an insufficient flow of blood, unable to satisfy the requirement of oxygen and nourishing substances of the cerebral tissue, defines ischemia. Cerebral ischemia is caused by an interruption of blood flow to the brain due to a clot and represents 87% of all stroke cases [3]. TOAST classification distinguishes five different subtypes of stroke [4]: (1) large artery atherosclerosis (LAAS); (2) cardioembolic infarct (CEI); (3) lacunar infarct (LAC); (4) stroke of other determined etiology (ODE); (5) stroke of undetermined etiology (UDE). Most of the strokes with ischemic origin are caused by atherosclerosis affecting the large artery and by embolism with cardiac genesis. Approximately 45% of ischemic strokes are provoked by a thrombus in a small or large artery [5,6] whereas cardioembolic stroke accounts for 14–30% of all cerebral infarctions [7,8]. Another important subtype is the lacunar one (15–25% of all ischemic strokes) [9,10]. Therefore, cardiac embolism, atherosclerosis of the cerebral circulation and occlusion of small vessels can be the cause of ischemic stroke. Among all of them, cardioembolic stroke has relevance for two reasons: first, cardiac embolism provokes the most serious strokes [11], secondarily, despite developing better therapies for dyslipidemia and arterial hypertension, embolism with cardiac origin represents a rising source of stroke in wealthy nations, for example, Canada [12]. The processes that take place in cells and molecules involved in brain injury, caused by ischemic stroke, have been widely examined.

The occlusion of a cerebral artery results in a deficiency of oxygen, glucose, lipids and, consequently, in necrosis of the cerebral parenchyma. Multiple mechanisms, including excitotoxicity, oxidative stress, and inflammation, have been considered to explain the brain injury caused by ischemia [13]. The ischemic neuronal damage causes a significant release of glutamate, which leads to excessive activation of NMDA receptors and a massive flow of Ca^2+^ into cells, causing their death due to excitotoxicity [14,15]. After the process of ischemia-reperfusion, damaged neurons and astrocytes produce reactive oxygen species (ROS), the same process depletes glutathione, one of the essential antioxidant agents that prevent reactive oxygen species-mediated DNA injury [16]. Critically damaged cells and remnants, even in no presence of microbes, cause the inflammation that takes place after ischemia-reperfusion injury [17,18]. Oxidative stress and the triggering of the inflammatory process contribute to the rupture of the blood–brain barrier allowing activated blood-borne immune cells, such as neutrophils and T cells, to reach the cerebral parenchyma and accumulate in the tissue involved in ischemia [19,20]. Following the accumulation of activated immune cells from the periphery, microglia in the brain become activated after the ischemic process due to the rise of extracellular ATP from the depolarization of neurons and glia and the consequent release through injured plasma membranes of dying cells [21]. Activated microglia secrete proinflammatory agents, such as cytokines, and develop phagocytic and major histocompatibility complex (MHC) class II-restricted antigen-presenting properties [22]. Microglia activation has beneficial effects because it promotes the production of growth factors, such as brain-derived neurotrophic factors, and the removal of dead tissue and debris after ischemia; however, the release of proinflammatory cytokines, such as tumor necrosis factor-α (TNF-α), reactive oxygen species, and nitric oxide following the activation of microglia is detrimental [23,24,25]. As cells die and brain tissue is injured, molecular danger signals further increase phlogosis by activating more microglia and infiltrating leukocytes in a feed-forward response releasing more cytokines with proinflammatory action. The increased expression of cytokines further promotes the expression of adhesion molecules on endothelial cells that provokes additional recruitment of leukocytes from the peripheral blood [20]. This process, that takes place after ischemia, leads to an increased neuronal cell death causing a larger infarcted area and a worse neurological outcome. Therefore, following the ischemic stroke, a local and systemic phlogosis takes place and plays a crucial function at all stages of the ischemic mechanism, starting from the cessation of blood flow to the late regenerative processes correlated with the repair of the tissues involved in ischemia. Neuroinflammation promotes further injury causing cell death, but at the same time has a beneficial function supporting recovery. This inflammatory response takes place during all subtypes of stroke, but it seems to be amplified in the cardioembolic one. It could, at least in part, explain the more critical neurological symptomatology and the worse outcomes that characterize cardioembolic stroke. In this review, we analyze the beneficial and harmful roles of neuroinflammation and the possible future therapeutic strategies to limit pathological responses. Here, we also focus on the peculiar inflammatory background of cardioembolic stroke by analyzing the interaction that connects heart and brain damage.

## 2. Neuroinflammation in Ischemic Stroke

**Figure 1:**
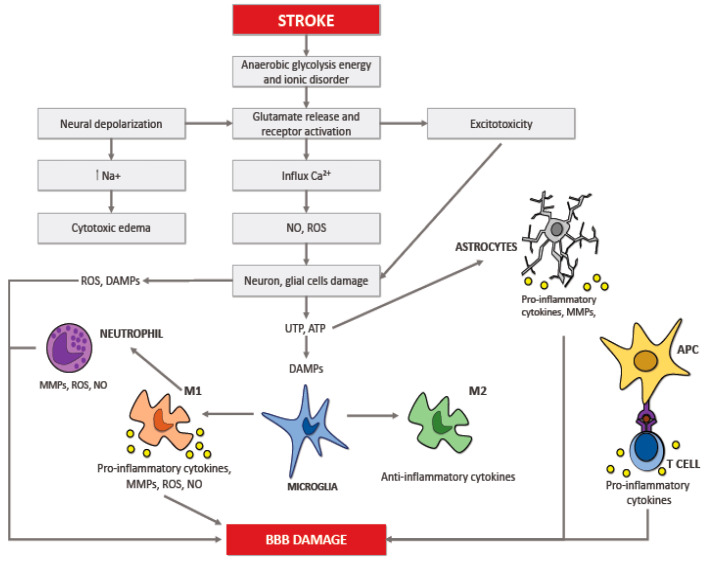
Pathomechanism of ischemic brain damage.

Numerous studies showed that the inflammatory reaction that occurs in the cerebral tissue takes place in multiple acute pathologies of the brain, including ischemic stroke; this response is called neuroinflammation [19,20]. The neuroinflammation process depends on the scene, period, and course of the neurological insult. The brain insult that follows ischemic stroke results in necrosis and apoptosis; all of this drives an inflammatory reaction controlled by the discharge of ROS, chemokines, and cytokines. This process springs up in the microcirculation and involves several cytotypes, such as innate immune cells (i.e., the microglia) and adaptive immune cells (i.e., lymphocytes) causing neuronal death (Figure 1) [23,24,25]. After that inflammation occurs in the brain, it intensifies the discharge of multiple cytokines in both damaged cerebral tissue and peripheral blood. These cytokines represent important mediators during the stroke-induced immuno-inflammatory reaction, that are involved in the progression of the cerebral infarct and influence disease severity and outcome [26,27,28,29,30].

## 3. Roles of Cytokines in Cerebral Ischemia

Cytokines are agents able to modulate the immune system; they have a crucial function in the process of cell activation, differentiation, and proliferation. Almost every nucleated cell can produce cytokines to modulate the communication between immune cytotypes such as monocytes/macrophages, B cells and T cells and, as a result, arrange immune reactions [27,28,29,30]. Cytokines with a proinflammatory action are involved in many processes that take place in the cerebral tissue where they can trigger endothelial cells, neurons, and glia directly. Due to this intricate and multistep pathway, they could increase cellular traffic or cause additional injury.

### 3.1. TNF-α

TNF-α was earlier named “differentiation factor” or “cachectin”; this protein takes part in almost all the immune and inflammatory processes. Mast cells, monocytes, T cells, neutrophils, keratinocytes, macrophages, and fibroblasts can produce this cytokine. We can observe TNF-α in a couple of distinct types: a transmembrane one (tmTNF-α), able to modulate inflammation locally by communication between cells, and another one that is soluble and biologically active (sTNF-α) [31]; the tumor necrosis factor-alpha converting enzyme (TACE) produces the active form. The soluble type operates systemically by promoting the phagocytosis of macrophages and their cytotoxic action; it also stimulates the production of IL-1 and IL-6, besides other cytokines. TNF, apart from its systemic work, has also a local one in the central nervous system. TNF-α performs its activities using binding receptors (TNFR-1 and TNFR-2) with different percentages of glycosylation and attraction for TNF-α. sTNF-α has more attraction for TNFR1, on the other hand, tmTNF-α for TNFR-2 and TNFR-1. Growing evidence suggests that TNF-α has a crucial function in the neuroimmunological development of stroke and has both a neurotoxic and a neuroprotective result in the ischemic brain [28,29,30]. Proinflammatory agents, such as interleukin (IL)-6, IL-1, and TNF-α, promote the developing of the harmful phlogosis that takes place in the brain after the ischemia [28,29,30]. Various studies [28,29,30], using first preclinical animal models and then collecting human samples from patients affected by cerebral ischemia, propose that TNF-α, which is present in the whole body and has a robust proinflammatory action, may have a role in the process that causes ischemic injury in the cerebral tissue.

Furthermore, there is an overexpression of TNF-α in the cerebral parenchyma following both the definitive [32] and the temporary occlusion of the middle cerebral artery (MCAO) [33]. TNF-α is one of the first cytokines to emerge in the context of the inflammatory reaction following ischemic injury in the brain and takes part in the stimulation of the inflammatory process in both cerebrospinal fluid and blood serum [34,35]. Indeed, TNF-α’s appearance in the brain following the damage induced by ischemia is quick and has a first peak in the early hours [1–3 h] and a successive one after more than 24–36 h [34,35]. In the literature, some reports indicate that TNF-α represents an accurate indicator that can be useful to define the beginning of the inflammatory reaction and a helpful one for the evaluation of the patient’s outcome [36]. A rising quantity of TNF-α can be seen in patients affected by a stroke just 6–12 h following the appearance of the symptomatology [37]. We can also observe that the quantity of TNF-α increases within 24 and 48 h following cerebral ischemia; the slight reduction, which takes place within 72 and 144 h after a stroke, is correlated with an improvement of the clinical conditions in patients during the acute stage of cerebral ischemia [38]. Additionally, TNF-α has a role in protecting the cerebral parenchyma from the damage caused by ischemic stroke [39]. Consequently, it appears to have a bivalent action in the cerebral inflammation that follows the first stages of ischemia; in fact, it plays a proinflammatory function during the first step of the inflammation in the central nervous system (CNS) and an immunosuppressive one during the chronic phase.

### 3.2. IL-1β

At least three proteins are part of the IL-1 group: IL-1α, IL-1β, and IL-1ra. These proteins are produced from different genes with a relevant homology and are involved in the development of many human pathologies, including cerebral ischemia. IL-1β is a cytokine with an essential role in regulating the immune system, and it can increase inflammation influencing nearly all the cytotypes. It seems that following systemic injuries, such as infections, or local ones, such as a stroke, IL-1β is the principal IL-1 agonist induced in the cerebral parenchyma in less than an hour during experimental damage in the brain caused by ischemia. IL-1β is made by macrophage/microglia and monocytes after the creation of the inflammasome [40]. Following cerebral ischemia, IL-1β can turn on the nuclear factor (NF)-κB through the stimulation of TLRs, after this process it can activate the part of genome correlated with chemokines, cytokines, and other factors with a proinflammatory role [41]. Following ischemic stroke, the microglia will be switched to the type able to stimulate inflammation, called M1, which expresses IL-1β, which is a proinflammatory cytokine with a neurotoxic effect. IL-1β can interact with the vascular endothelium increasing the adherence of the leukocytes and promoting the appearance of edema [42]. The cerebral damage induced by MCAO was significantly lower in IL-1β knockout mice [43]. Furthermore, the cerebral injury appeared to be more significant in rats with previous administrations of IL-1β [44]. Multiple studies report that we can observe the decreasing of the area of the brain damaged by ischemia, preserving neurological function, by inhibiting the IL-1 receptor 1, which can bind to both IL-1 isoforms and is identified in multiple cytotypes [45]. Therefore, this evidence suggests considering IL-1βa as a crucial contributor to ischemic brain damage. IL-1β, when tied up with its related receptor (IL-1R), is capable of stimulating the increase of NF-κB pathways IL-1R-dependent. However, when the concentration of IL-1β is over a determined cutoff, the expression of the antagonist of IL-1 receptor (IL-1Ra) is stimulated. The equilibrium of IL-1β and its antagonist, IL-1Ra, has more relevance than just IL-1β itself because of its global effect and role [46]. Thus, this balance could be a useful marker of the patient’s potential outcome after cerebral ischemia. However, not much clinical research has shown their possible application as biomarkers in the context of ischemic stroke. In a study, IL-1β levels were correlated with a poor prognosis and reduced long-term functional recovery [47], on the other hand, IL-1Ra levels seemed to be related to a higher risk of infections after the ischemic stroke [48].

### 3.3. IL-6

As a reaction after a cerebral injury, different cytotypes, for example astrocytes, leukocytes, endothelial and microglial cells, can produce IL-6. This cytokine makes hepatic cell produce fibrinogen, CRP (C-reactive protein), and other acute-phase proteins (APPs). IL-6 turns these proteins on and promotes the transcription of various genes by taking part in the process of phosphorylation of the NF-IL-6 transcription factor [49]. To be produced, IL-6 needs IL-1 and TNF capable of activating fibroblasts, endothelial cells, and keratinocytes and increasing the expression of IL-6. IL-6 is one of the proinflammatory proteins that play many crucial roles in the cerebral parenchyma with results that can be damaging and helpful at the same time. Many other molecules, for example, prostaglandins, TNF-α, IL-1, and IL-4, can influence the secretion of this protein; this fact makes us think that the whole process of inflammation can be related to the expression of multiple cytokines. In recent years, many pieces of research tried to explain the function of interleukins in the pathophysiology and evolution of cerebral ischemia. Even though IL-6 is a proinflammatory protein, researchers proposed that it has an essential function during an ischemic stroke because of its proinflammatory properties in the first step of ischemia, but also playing a neurotrophic role in the successive stages [50]. IL-6 is a verified indicator of inflammation during stroke; although the concentration of IL-6 in healthy condition is relatively modest, multiple studies showed that it increases significantly in serum in no more than a few hours following the beginning of the pathology; this peak persisted up to 90 days following the cerebral ischemia [51,52].

On the other hand, in a rat model of cerebral ischemia, the damage due to stroke was meaningfully reduced after giving recombinant human IL-6 [52]. Furthermore, Sotgiu et al. [37] reported an inverse relationship between the size of the brain affected by ischemia and the expression of IL-6. The group deduced that IL-6 also has a neuroprotective role in the cascade of inflammation during an ischemic stroke, not just a damaging one. Thus, this evidence suggests a possible bivalent role of IL-6 into the ischemic brain.

### 3.4. IFN-γ

We can distinguish two different types of cytokines in the IFN group. Type I IFNs represents the most substantial class. IFN-α, -β, -ε, -κ, and -ω are part of this type which presents a certain homology in different genes, furthermore many cytotypes can synthesize this class. The second type comprises just IFN-γ that is a cytokine capable of arousing and modulating a significant part of the immune responses [53]. Different cytotypes can produce IFN-γ, mainly monocytes, natural killer (NK) cells, macrophages, B-lymphocytes, dendritic cells, and T cells. IFN-γ represents one of the central modulators of the immune system; it is involved in the first steps of the response against many pathogens. Additionally, IFN-γ can modulate the immune system stimulating the expression of class I and II MHC molecules through T cells and macrophages; this process encourages the process of antigen presentation. The heterodimeric receptor (IFN-γR) situated on the cell’s surface mediates the cellular response to IFN-γ activating downstream signal transduction cascades, ultimately resulting in the regulation of gene expression. Multiple downstream signaling pathways, especially the one related to the Janus kinase (JAK) and the other one modulated by the activator of transcription (STAT), can be activated by IFN-γ after the connection with its receptor [54]. Therefore, thanks to these properties, IFN-γ could be involved in the mechanism responsible for the atherogenesis. A growing body of evidence has reported higher expression of IFN-γ in atherosclerotic lesions, suggesting its crucial function in the process of atherogenesis [55]. Therefore, it can also take part in the pathogenesis of ischemic stroke, one of the most atherosclerotic pathologies. Following an ischemic brain insult, we can observe the stimulation of CD4^+^ T cells with specific MHC class II capable of passing through the blood–brain barrier (BBB) reaching the CNS easily [56]. Therefore, CD4^+^ cells may be excited by microglia, and then follow two possible ways: become either T helper 1 cells (Th1) with the function to produce cytokines able to promote phlogosis, such as IFN-γ, TNF-α, and IL-6, or T helper 2 cells (Th2) capable of supporting the immune response mediated by antibodies producing IL-4, IL-5, IL-10, IL-13 [57]. It seems that IFN-γ can modulate the polarization of microglia actively. It was observed that the polarization of microglia into M1 phenotype is promoted by IFN-γ produced by Th1 cells; the M1 type is responsible for a proinflammatory reaction and can produce proinflammatory cytokines and oxidative metabolites.

## 4. Role of Anti-Inflammatory Cytokines

The unevenness between inflammation and the process against phlogosis seems to have a critical function in the development of cerebral injury due to ischemia [58]. IL-10 is a protein with anti-inflammatory properties whose gene is placed on chromosome 1. Monocytes are the primary producers of this protein. Still, it can be secreted by other cytotypes, such as Th2 lymphocytes, a specific subtype of T and B cells, CD4 CD25 Foxp3 regulatory T cells, and mastocytes [58]. IL-10 is critically involved in containing the cerebral damage during cerebral ischemia; in fact, it can suppress the excessive secretion of proinflammatory cytokines. Furthermore, research verified that the ischemic cerebral area could be decreased by the effect of the IL-10 [58], on the other hand, mice lacking this cytokine presented a wider damaged area by ischemia after MCAO [59]. TGF-β is a growth factor present in the whole human body. All the proper receptors have equal affinity for the three different isoforms which are TGF-β1, TGF-β2, and TGF-β3; the last one has a role in the process of survival of neurons and promotes the recovery of the cerebral parenchyma. TGF-β can inhibit neutrophils and astrocytes [60], so indirectly reduces the production of proinflammatory cytokines with harmful effects on the cerebral parenchyma after an ischemic stroke. Following cerebral ischemia, the inflammation can be modulated by TGF-β produced by activated M2 macrophage, promoting in this way the recovery from the ischemic insult [61]. TGF-β can inhibit the microglia cells and consequently reduce the potential damage caused by this cytotype. In the end, we can suppose that TGF-β is capable of decreasing the inflammation protecting the cerebral tissue in the context of cerebral ischemia. IL-4 is a cytokine capable of regulating the immune system and the development of phlogosis. It plays a vital role during Th2 cell differentiation [62] and can polarize macrophages/microglia toward the M2 type, which has anti-inflammatory properties [63]. M2 macrophages/microglia produce multiple mediators playing an anti-inflammatory role; they also can secrete numerous factors capable of supporting the recovery of the cerebral tissue. They can also reduce the phlogosis promoting the phagocytosis of dead cells and the proteolysis of specific proteins, indirectly supporting the rehabilitation of the brain [64]. It seems reasonable to hypothesize that this cytokine could encourage cerebral recovery and hence have a crucial role in the development of future therapies.

## 5. Pro and Anti-Inflammatory Cytokine Interplay in Ischemic Stroke

The presence of phlogosis following acute cerebral ischemia is relatively well established. Cytokines such as IL-6 and TNF-α seem to be crucial mediators of this inflammation. Increased release of proinflammatory cytokines has been reported in experimental models of cerebral ischemia as well as in subjects affected by acute stroke [65,66] and could be correlated with a larger ischemic area and poorer outcome. An increased concentration of IL-6 in both cerebrospinal fluid (CSF) and serum have been linked with aggravating neurological symptomatology, more extensive infarct size, and worse prognosis [67]. IL-6 can induce the secretion of prostaglandin E2 in the cerebral parenchyma; this prostaglandin causes a rise of body temperature by operating on the hypothalamus. Several human and experimental stroke studies have reported that fever is associated with increased infarct size and poor outcome; thus, the early and prolonged elevation of IL-6 in the CSF and blood most likely correlates with an increased chance of elevated temperature that stimulates inflammation and the consequent injury of the cerebral parenchyma after stroke. Moreover, increased levels of TNF-α in the serum and CSF of subjects affected by stroke are associated with a worsening of neurological symptoms, increased infarct area, and poorer outcome at 3 months in research including severe strokes and patients with impaired white matter [68].

On the other hand, there are molecules with anti-inflammatory function, such as IL-10 and IL-4, which counteract the proinflammatory cytokines. In physiological conditions, there is a delicate balance between proinflammatory and anti-inflammatory cytokines; the exact process of this interaction and the correlation with the clinical outcome of subjects affected by ischemic stroke is still unclear. Nevertheless, this balance is interrupted in the early stage of acute ischemic stroke. On this purpose, Basic Kes et al. performed research [69] to investigate the different concentration of IL-6, TNF-α, and IL-10 of acute ischemic stroke patients and control subjects and the possible correlation of plasma cytokine levels with stroke severity at admission and the outcome. The authors reported that proinflammatory IL-6 measured 12 h after the onset of cerebral ischemia has risen in stroke patients relative to controls; this increase was correlated with more severe neurological symptomatology and worse outcome.

In contrast, anti-inflammatory IL-10 was reduced and associated with a better outcome. This finding confirms the presence of a close interaction between proinflammatory and anti-inflammatory molecules during the first steps of stroke; the loss of this balance in favor of inflammation causes a more serious neurological symptomatology. However, at the moment, we cannot determine precisely the processes that modulate this interaction. Ischemic stroke represents a heterogeneous pathology and multiple factors, including stroke severity and location, age, comorbidities, systemic inflammation before the stroke, and lastly, genetic variations in interleukin genes, may influence the alterations in the serum level of inflammatory markers during the acute phase of cerebral ischemia.

## 6. Recruitment of Inflammatory Cells in Ischemic Brain Injury

The involvement of other cells apart from neurons in the development of cerebral damage induced by ischemia has been investigated in multiple studies. Furthermore, it is plausible to consider the existence of a functional “neurovascular unit” made by the set of neuronal, glial, and vascular cells. After a cerebral ischemia inflammation starts to develop, it begins with the expression of proinflammatory factors and involves various cell types: first of all we can observe the switching on of the glial cell resident in the brain. Leucocytes, followed by monocytes and other cells with immune functions, come into the cerebral tissue. This mechanism could cause cerebral damage induced by ischemia, characterized by the formation of edema and the progressive death of neurons. Various cytotypes involved in this process may have a detrimental or beneficial effect depending on the stage of the development of ischemia, early or delayed; consequently, we have to reflect on the timing of their involvement in the cerebral area affected by ischemia.

### 6.1. Microglia

**Figure 2:**
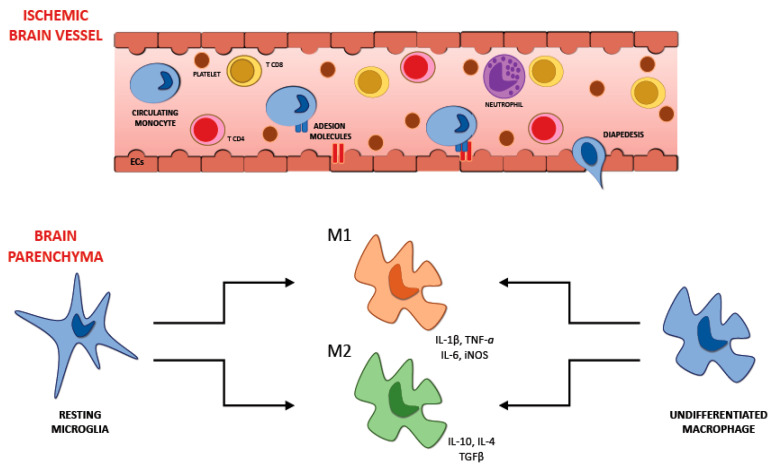
Activated microglia have two activation phenotypes: classically activated (M1) and alternatively activated (M2). M1 microglia are considered as proinflammatory and produce proinflammatory cytokines and oxidative metabolites such as IL-1β, TNF-α, IL-6, and nitric oxide. M2 microglia contribute to recovery after brain injury, are activated by IL-4, and express anti-inflammatory mediators, such as IL-10, IL-4, and TGF-β. IL: interleukin; TNF: tumor necrosis factor; TGF: transforming growth factor.

Microglia are part of the cells resident in the brain; they belong to the innate immune system and constitute the 5–20% of all the glial cells. These cells are stimulated following cerebral ischemia and, as a result, morphological and phenotypical changes can be observed [70]. The cells of microglia in the cerebral tissue are one of the first cytotypes to take part in the immune response. They are activated just a few minutes after the beginning of the ischemic injury, increasing their number in the next days, with the climax 10 days following the transient focal ischemia in the cerebral parenchyma [71]. On the other hand, the macrophages that come from the systemic circulation take part in the inflammatory mechanism with some postponement. We can notice their presence in the cerebral tissue 4 days after the beginning of the ischemia, with the peak after 7 days and then their concentration reduces [71]. Microglia assume their activated phenotype after the ischemic insult, exposing some ramifications and taking an amoeboid aspect, assuming the form of the macrophages once turned on. After this process microglia cells look like macrophages not only in the appearance but also in their abilities, they can expose antigens, release various cytokines, and secrete metalloproteinases of the extracellular matrix capable of harming the BBB and consequently increasing its permeability. This process could make the early passage of the leukocytes from the systemic circulation to the cerebral parenchyma more accessible, resulting in an increased concentration of proinflammatory factors capable of worsening the injury due to the ischemia. Once turned on, microglia can assume two different phenotypes: the classical activated (M1) and the alternative one (M2) [22] (Figure 2). The M1 phenotype has a proinflammatory function and can release cytokines and substances with oxidative properties, such as nitric oxide, TNF, IL-6, and IL-1β [72]. M2 phenotype plays a beneficial action promoting the healing of the cerebral parenchyma after the ischemic insult and releasing anti-inflammatory agents such as IL-4 and IL-10; it also secretes multiple factors with neurotrophic properties capable of averting inflammation.

During the cerebral ischemia, we can observe that in the first steps the primary phenotype of both resident microglia and macrophages that come from the systemic circulation is the M2 one. Around the areas directly involved in ischemia, the M1 phenotype becomes more relevant in the following phases. Thus, the neurons located in the regions affected by stroke can stimulate the microglia and macrophages to switch into the M2 phenotype [72]. Thinking about the entirely different action of the two different phenotypes that microglia can assume during cerebral ischemia, developing a therapeutic strategy able to suppress the morphological transformation and encourage the benefits of microglia appears to be crucial.

Although several works in the literature use the terminology of “macrophage polarization”, most commonly into the M1 and M2 phenotypes, to date, the presence of microglial M1/M2 polarization has been subject to a fierce debate [73]. Indeed, some authors doubt the real existence of polarization of microglia. Profiling of microglia genome from mice affected by many diseases [74,75] did not manage to demonstrate evidence for M1/M2 polarization. Moreover, comprehensive profiling of 299 human macrophage transcriptomes derived from 29 in vitro exposure conditions followed by coregulation analysis and hierarchical clustering on Pearson correlation matrices revealed a substantial variety of expression profiles [76]. M1 and M2 expression profiles lay near the baseline, relative to those induced by stimuli such as fatty acids, cytokine cocktails, and prostaglandins. The M1 and M2 expression profiles presented no signs of organizing value, and it was clear that other transcriptional profiles failed to line up between these two states, as would be predicted from a continuum model of polarization.

Therefore, the adoption of this schema may have been undertaken trying to simplify data interpretation when the ontogeny and functional significance of microglia had not yet been defined.

### 6.2. Astrocytes

Another resident cytotype in the brain is astrocytes; they are deeply involved in preserving the correct functioning of the CNS. They regulate the balance of water and various ions, secrete some neurotrophic factors, remove neuromediators after any synapses, transport multiple products and wastes of the cellular metabolism. Astrocytes even influence the structure of the BBB [77]. During homeostasis, astrocytes can transform the excessive glutamate outside the cells into glutamine to be used again by neurons; still, following cerebral damage, this ability of astrocytes might be weakened [77]. After ischemia, glial cells and neurons release cytokines able to cause reactive hyperplasia of astrocytes provoking the secretion of agents (nestin, vimentin, monocyte chemotactic protein-1, IL-1β, and glial fibrillary acidic protein (GFAP)) ready to promote reactive gliosis and the formation of scars [78,79]. Following the cerebral ischemic damage, the malfunction of the Na^+^-K^+^ pump causes the expansion of the astrocytes, increasing the intracerebral pressure and reducing the perfusion of the cerebral parenchyma [80]. After their activation, astrocytes can release the matrix proteinase-2 able to damage the extracellular matrix [81]; they can also promote the presence of ephrine-A5 in the cerebral area injured by ischemia, hampering the axonal sprouting [82].

### 6.3. Neutrophils

Apart from macrophages from the systemic circulation and microglia, the quantity of neutrophils in the brain affected by ischemia is one of the most significant. Their appearance is quite early, they reach the cerebral parenchyma 30 min to a few hours after the damage due to ischemia, with a peak in the next 3 days and a gradual reduction [83]. Still, we can notice their presence until 15 days after the ischemic injury. This cytotype can display molecules able to adhere to the endothelium just 15 min after the ischemic damage [84]. In the next 6–8 h, we can see neutrophils surrounding the blood vessels of the brain and reaching the cerebral parenchyma [85,86]. The exact function played by neutrophils in the development of ischemic damage is still unclear. Still, there are some hypothetical mechanisms: the obstruction of blood flow in the cerebral circulation (CBF) by secreting vasoconstrictive factors or the extreme release of proinflammatory agents, ROS, and enzymes with hydrolytic properties [87]. Additionally, neutrophils are capable of producing MMP-9, which is a protease able to damage the basal lamina, the BBB, and harm the brain tissue causing edema and hemorrhagic transformation of ischemic stroke [88]. Infarct volume and functional deficits are proportional to neutrophils increase [83]. On the other hand, lymphocyte numbers decline after cerebral ischemia increasing the ratio between neutrophils and lymphocytes, which is directly correlated to the extension of the area affected by ischemia and the consequent risk of death [89].

### 6.4. T Lymphocytes

Differently from neutrophils, T cells take part in the later phases of the cerebral ischemia. These leukocytes infiltrate within 3 days the peripheral zone surrounding the lesion by sparing the center, often in proximity to the arterial circulation; their number rises after 3 days with the maximum after a week, then reduce after another one [90]. Different studies tried to find out the role of the various T cells types during cerebral ischemia. Depending on the different actions, it is possible to identify three types of T cells, the cytotoxic (CD8^+^), the helper (CD4^+^), and the regulatory ones (Tregs). The distinct character and role of these T cells are defined by the several cytokines secreted, and by the markers exposed on their surface. The essential purpose of CD4^+^ and CD8^+^ T cells in developing phlogosis and thrombosis, with consequently increased cerebral injury and worsened neurological deficit by influencing the same pathways, was reported by various research on T cell-deficient mice [29,30,32].

Tregs exposes CD25 and Foxp3, a transcription agent, on their surface; moreover, this subtype represents the 10% of all the CD4^+^ cells. Various research teams are focusing on the possibility of taking advantage of their beneficial action for the brain by controlling their functioning. Numerous studies [91,92] indicate IL-10 and Treg cells as essential neuroprotective factors from inflammatory postischemic brain damage. They seem to counteract the destructive action of IFN-γ, TNF-α, and other cytokines with a proinflammatory function. Additionally, Treg cells are probably able to interfere with the coming of the immune system in the cerebral parenchyma, which represents a fundamental trigger of the phlogosis following the stroke. Furthermore, immunodepletion of Treg in mice, mediated by CD25 specific antibody, exacerbated tissue loss and was associated with a more serious neurological symptomatology 7 days following ischemia provoked by MCAO [92]. However, Treg cell therapy has already its possible strategies under study; one of the options is to use their suppressive action once isolated, purified, and expanded. In the family of T cells, we can recognize another subset, the γδ T cells, which represent 5% of the T cells population and has some peculiarities. This subtype has a particular T cell receptor (TCR), usually composed by α and β glycoprotein chains, made by one γ chain and a δ one. Even this cytotype seems to take part in the development of cerebral damage caused by ischemia. Various research showed that mice lacking TCR-γδ had a remarkable reduction of the cerebral tissue involved in ischemia; the same result was observed in mice after the administration of antibodies against this receptor [93]. These findings could reveal new possible therapeutic strategies for reducing the phlogosis that takes place in the cerebral parenchyma after the stroke. We can notice a subtype of T cells that do not express CD28, which has a stimulatory action.

**Figure 3:**
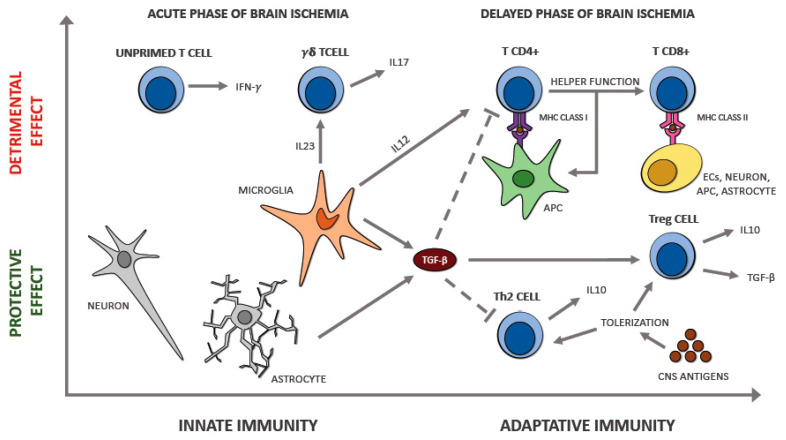
T cells involvement in acute ischemic stroke.

CD4^+^ CD28null T cells, the name of this atypical cytotype of T helper, are almost ubiquitous in normal conditions and are the 0.1–2.5% of the CD4^+^ T cells located in the blood. They can release a significant quantity of IFN-γ and TNF-α [94,95]; consequently, they have a proinflammatory action (Figure 3). Moreover, this cytotype has a damaging effect by producing granzyme A, granzyme B, and perforin that are usually released by CD8^+^ T cell and NK cells, not by T CD4^+^. CD4^+^ CD28null T cells are resistant to apoptotic signals and the suppressive action made by Tregs; moreover, they cannot stimulate B cells to produce antibodies because of the absence of CD40 and the CD28 receptor [96]. Nowik et al. [97] hypothesized that this cytotype might play a damaging function during cerebral ischemia when an atherosclerotic background is present. They reported that these T cells are involved in some processes able to increase the risk of acute ischemic stroke, denying that their appearance is just a result of the cerebral ischemia. This finding opposes the hypothesis of an increase in the number of these cells after the cerebral ischemic insult. The authors reported that the quantity of CD4^+^ CD28null T cells was similar in two groups (a group in the first step of cerebral ischemia and another one with no story of ischemic stroke but with either diabetes or hypertension) but was significantly higher than in controls. This fact may suggest a possible role played by these cells, together with other known risk factors, in stimulating the atherosclerotic process and weakening the plaque. Furthermore, this research showed that the intensity of neurological damage, evaluated by the characteristics of the cerebral areas affected by ischemia observed by brain CT scan performed within 24 h from the beginning of the ischemic process, is not associated with the percentage of lymphocytes.

Z.G. Nadareishvili et al. [98] observed the potential increase in the number of CD4^+^ CD28null lymphocytes in patients affected by multiple strokes or who died because of this pathology. The authors focused on the quantity of this cytotype in the circulating blood monitoring 106 patients in the 48 h successive to the onset of the neurological symptomatology; they also performed a 1-year-long follow-up. They found out a correspondence between the chance of death due to stroke or new episodes and the concentration of these cells, proposing CD4^+^ CD28null lymphocytes as a possible indicator for patients at high risk of new ischemic events or death. A possible explanation of this evidence is the capacity of these cells to injure blood vessels and the cerebral parenchyma promoting phlogosis. Moreover, this subtype of T cells could be a marker of deficient adaptive immunity. Tuttolomondo et al. performed research [99] with the purpose to correlate the concentration of CD28null cells in the blood and the different type of cerebral ischemia according to the TOAST classification. The authors also wanted to verify a possible association with the severity of the symptoms measured with validated scores, evaluating this cytotype as a potential predictive value for the diagnosis and the definition of the subtype of cerebral ischemia. In total, 98 consecutive subjects with a diagnosis of ischemic stroke took part in the study; 66 hospitalized patients with no acute cerebral ischemia were registered too as controls. The authors have recounted that the concentration of CD4^+^ cells and CD28null cells were significantly higher in patients affected by acute cerebral ischemia than in controls. Besides, subjects affected by cardioembolic stroke showed a blood quantity of these cytotypes relevantly increased compared to the other ones. They also reported a relevant correlation between CD28null cells outer percentage and some markers of stroke severity, such as the SSS score and the NIHSS, considered as evaluators of the degree and type of neurological deficit during the acute phase. The results of this study hypothesize that, in the setting of acute cerebral ischemia, a higher percentage of peripheral CD4^+^ CD28null cells might be associated with a more profound brain injury. Killer immunoglobulin-like receptors (KIRs) interact with human leukocyte antigen (HLA) class I molecules influencing the ability to recognize targets of NK cells and T cells. Recent evidence [100] suggests the T cell implication in the development of the acute pathologies correlated to atherosclerosis, implying that the KIRs panel of a subject could modulate the functioning of inflammatory cells during critical cardiovascular events. On this basis, Tuttolomondo et al. performed research [101] to evaluate a possible link between the susceptibility to cerebral ischemia and the genetic setting of the immune system in correlation with the frequency of the KIR genes and HLA alleles. Between November 2013 and February 2016, 116 consecutive patients with acute ischemic stroke were recruited. As healthy controls, 66 subjects without acute ischemic stroke were enrolled. Patients affected by acute cerebral ischemia and control patients were genotyped for the existence of KIR genes and of the three main KIR ligand groups, HLA-C1, HLA-C2, and HLA-Bw4, both HLA-B and HLA-A loci. The authors found an increased expression of activating “proinflammatory” KIR genes in subjects with ischemic stroke, which could justify the massive development of phlogosis during the acute phase of the stroke. Indeed, the previous finding [99] of an increased peripheral blood percentage of CD28null cells in patients affected by cerebral ischemia could be associated with an amplified expression of 2S2 and 2DS4, KIR genes with a proinflammatory activity. The authors concluded that KIR genes, modulating the expression of KIRs, could promote the expression of cytokines and the development of inflammation mediated by some cytotypes such as NK cells and other T cells.

## 7. The Interplay between Immune Cell Populations in Ischemic Stroke

There is a tight connection between the central nervous system and the immune system through complex communicating networks. The immune system monitors the brain functioning and reacts when cerebral homeostasis is altered because of injuries or diseases. Stroke promotes strong phlogosis involving the local production of cytokines, such as TNF-α, by various cytotypes in the brain, including human neurons, activated glial and endothelial cells, with consequent blood–brain barrier detriment and infiltration of multiple types of leukocytes after a determined interval of time. These cytotypes can play a neuro-damaging or neuroprotective function, and the severity of the cerebral injury is strictly correlated with the balance between these two possible functions.

The interaction between the various cytotypes of the immune system during the acute phase of ischemic stroke is an intricate mechanism regulated by several factors. The ischemic stroke is a complex pathology, and numerous factors, such as the severity of the ischemic lesion, the location of the stroke, age, and comorbidities, can affect not only the interaction but also the equilibrium between the cytotypes in the necrotic cerebral parenchyma. All these factors can affect the local cytokine secretion, which has a crucial function in modulating the interactions between the various immune cells involved. For example, severe ischemic injury and an increased proinflammatory milieu with massive IL-6 and TNF-α release may induce an increased neutrophil recall in necrotic cerebral tissue. Neutrophils are considered harmful since compelling evidence correlated this cytotype with blood–brain barrier breakdown and brain injury. Additionally, an increased blood neutrophil count is associated with larger infarct areas in subjects affected by cerebral ischemia [102]. Furthermore, the role of microglia and monocytes/macrophages during cerebral ischemia depends on the M1/M2 polarization status. The presence of specific cytokines in the local milieu (i.e., M1: IFN-γ, M2: TGF-β, IL-10) influences the polarization to one of the two possible phenotypes. The prevalence of the M1 phenotype is correlated with more severe ischemic damage, activation of the hypoxia-inducible factor-1 (HIF-1), and increased anaerobic glycolysis. Polarization of the microglia to the M1 phenotype and the consequent increased production of IL-23 promotes the recruitment and stimulation of γδ T cells, a subset of unconventional innate T cells with a different T cell receptor that could play a detrimental function during acute ischemic stroke. Growing evidence supports that γδ T cells are pathogenic in experimental cerebral ischemia/reperfusion models by secreting IL-17 and stimulating phlogosis [103]. On the other hand, the prevalence of a local anti-inflammatory milieu promoted by the secretion of IL-10 and TGF-β encourages the polarization of the microglia to the anti-inflammatory M2 phenotype, which has a neuroprotective function. Furthermore, the release of these anti-inflammatory cytokines promotes the recruitment of regulatory lymphocytes that play immunomodulatory and immunosuppressor functions in the injured cerebral parenchyma. Indeed, consistent evidence supports the beneficial functions of Tregs in an experimental cerebral ischemia model [80].

The interaction between the different populations of immune cells can be explained not only by the cytokine environment. Probably, these interactions are more intricate and comprise intercellular crosstalks by mechanisms that are not entirely understood. Therefore, the interactions between the immune cytotypes involved in ischemic stroke are complicated, and the equilibrium between neuroprotective and neurodegenerative actions is influenced by several factors, compatibly with the heterogeneous essence of the ischemic stroke.

## 8. The Interaction between Brain and Heart: Cardiac Complications after Stroke

It is common to observe cardiac injury in patients affected by cerebrovascular disease [104]. In 1947, Byer and colleagues first reported that cerebral vascular disease could provoke myocardial harm and arrhythmia. Typically, stroke induces neurovascular uncoupling and interferes with cerebral auto-regulation; this process makes cerebral blood flow directly dependent upon cardiac function [105]. Several interplays take place among the various forms of cerebrovascular and cardiovascular pathologies. Arrhythmias, ischemia like electrocardiographic (ECG) alterations, and myocardial injury are frequently observed in patients affected by acute cerebral ischemia, even in the absence of primary heart disease, which supports the hypothesis of CNS source as the cause of these cardiac abnormalities. Bilt et al. reported that cardiac dysfunction is correlated with premature cerebral ischemia and poor outcome after subarachnoid hemorrhage (SAH) with a consequent increased risk of death by a meta-analysis including 25 studies with a total of 2690 patients [106]. Consequently, it is reasonable to hypothesize that a causal relationship between brain damage and heart dysfunction exists.

### 8.1. Myocardial Injury as a Consequence of Brain Damage

The second leading reason for death after ischemic stroke is cardiovascular complications [107]. Broadly, stroke-induced cardiac damage could provoke lifelong diseases such as heart failure, or mild and recoverable injury such as neurogenic stress cardiomyopathy (NSC) and Takotsubo cardiomyopathy. The cardiac dysfunction that takes place during the acute phase of cerebral ischemia resolves in the next weeks alongside the improvement of neurological function. Treating the ischemic stroke is more important than treating heart dysfunction. NSC is diagnosed by observing reduced left ventricular ejection fraction (LVEF), ventricular wall motion abnormalities, and elevated serum cardiac enzymes. Takotsubo cardiomyopathy has similar symptomatology of NSC and shares the transient nature, but it is induced by psychological stress with no physical damage to the cerebral parenchyma. The telltale sign of Takotsubo cardiomyopathy is apical ballooning, provoked by a weakening of the heart’s muscular cells. Still, Takotsubo cardiomyopathy and NSC may be the cause of ventricular wall motion abnormalities in other regions of the heart [108]. Many studies focus on the impaired systolic function of the ventricle, which results in reduced ejection fraction, but it seems that the diastolic phase of the ventricle is also involved in NSC.

### 8.2. Ischemic Stroke Caused Cardiac Injury

The risk of cardiac complications is proportional to the severity of the cerebral ischemia and neurological symptomatology [107]. Likewise, when cardiac function is impaired as a consequence of severe acute ischemic stroke, it represents a predictor of worse functional prognosis and secondary complications [109]. Clinically relevant secondary complications, such as vasospasm and pulmonary edema, require immediate treatment. Recent evidence suggests that cerebral ischemia can induce cardiac dysfunction even with no risk factors and pre-existing heart disease [110]. During the first 3 months following acute ischemic stroke, 19% of patients suffer from at least one severe cardiac adverse event; 28.5% have left ventricular ejection fraction less than 50%, and 13–29% have systolic dysfunction [111]. Approximately in 67% of subjects affected by acute ischemic stroke, it is possible to observe ECG abnormalities suggestive of ischemia or arrhythmia in the first 24 h after the cerebrovascular accident [112]. A common reason for death after acute ischemic stroke is represented by cardiac arrhythmia. About 88% of stroke patients with injured insular cortex in the right cerebral hemisphere develop a myocardial injury in the weeks following ischemic stroke. Stroke, in particular the lacunar subtype, also induces heart problems in up to 70% of patients, with clinical manifestations such as ECG changes, reduced LVEF, ventricular wall motion abnormalities, and increases in serum cardiac enzymes [113]. However, the mechanism involved in the cardiac dysfunction associated with lacunar stroke is not well established.

### 8.3. Mechanisms Underlying Brain–Heart Interaction

The mechanisms that regulate brain–heart interaction are various and include catecholamine surge, sympathetic and parasympathetic regulation after stroke, and the phlogosis induced by the cerebral injury. The most acknowledged mechanism of brain–heart interaction is the catecholamine surge theory. This theory has been closely correlated with heart damage after physical and emotional stressors; in fact, catecholamine surge can induce cardiac hypertrophy or myocardial ischemia [114]. The autonomic nervous system modulates the release of catecholamines from the adrenal secreting cells. Brain damage can lead to an increased sympathetic tone with further increase in catecholamine secretion [115]. Neurologic damage promotes excessive circulation of catecholamines and massive adrenergic release from myocardial nerve endings. The myocardium adjacent to the nerve is injured [116]. The sympathetic nerve can directly release catecholamines with consequent cardiomyocyte toxicity. Long-term rise of serum catecholamine causes cardiotoxicity and may trigger edema in hypokinetic areas, transient fibrosis, phlogosis, and contraction band necrosis [117]. Experimental animal studies have proved an increase in plasma catecholamine levels after cerebral ischemia that was proportional to the presence of myocardial damage and cardiac injury [118]. Increased catecholamine concentration is associated with QT-interval prolongation and myocardial damage after SAH, while the stimulation of hypothalamus leads to ECG variations even with no myocardial damage [119]. The forebrain has a crucial function in modulating of the autonomic nervous system in subjects affected by ischemic and hemorrhagic stroke. Arousing the anterior cingulate gyrus and the orbital surface of the frontal lobe influences heart rate and blood pressure [120].

The insular cortex is considered a crucial component of the central autonomic network. When the insular cortex is affected by ischemia, there is an increased risk of cardiac complication, and it is possible to observe blood pressure variations, cardiac arrhythmias, and myocytolysis [121]. The location of the ischemic lesion also influences the possible cardiac dysfunction following a stroke, and some studies have reported cardiac impairment after an ischemic injury to both left and right insular cortex [122]. It was reported that the right hemisphere has a dominant function in controlling sympathetic activity, while the parasympathetic one is mainly controlled by the opposite hemisphere [123]. Therefore, a right insular injury provokes a reduction in sympathetic tone and with a consequent excessive parasympathetic activity [123]. Right insular lesions are correlated with increased mortality at an early stage compared with other possible sites of cerebral ischemia. Cerebral ischemia in the left hemisphere is associated with reduced incidence of arrhythmias; on the other hand, there is an increased chance of adverse cardiac prognosis, increased long term mortality and decreased cardiac wall motion compared to stroke in other sites [124]. During the first stages of SAH, it was reported an elevated activity of the sympathetic nervous system that leads to myocardial injury and promotes the development of cardiac dysfunction [125]. Consequently, intensive heart monitoring in subjects affected by insular cortical injury appears to be crucial.

Finally, it was well established that phlogosis takes part in neuronal injury due to ischemia. There are also some findings of an association between inflammation and sympathetic activation in ischemic and hemorrhagic stroke [126]. The proinflammatory cytokines secreted by injured neurons and glial cells make the posterior hypothalamus increase sympathetic output with consequent higher serum catecholamine concentration [127]. Bilt et al. reported that catecholaminergic stress following brain hemorrhage was associated with infiltration of inflammatory cytotypes into the heart with consequent cardiac injury [128]. Inflamed myocardium is characterized by the presence of neutrophils along with thrombi. The secretion of catecholamine due to parasympathetic dysfunction modulates phlogosis that promotes myocardial dysfunction, thrombi formation, and cardiomyocyte death. Therefore, inflammation could play a vital function linking cerebral and cardiac injury after ischemic stroke and SAH.

## 9. Cardioembolic Stroke

Ischemic stroke affects 26 million subjects all over the world each year; it is a significant reason for permanent physical disability and the second cause of death in the world. Cardiac embolism represents an increasing percentage of cerebral ischemia, and it is plausible to think that it will rise largely in the future. This kind of stroke is caused by an embolus formed in the heart that occludes a cerebral artery. Not all the possible cardiac origins have the same risk to cause an ischemic stroke; in fact, some of them have a high risk to provoke embolism, others a medium risk instead. It is necessary to identify at least one cardiac cause of embolism to establish that cardioembolism produced the cerebral ischemia.

### 9.1. Risk Factors for Cardioembolic Stroke

#### 9.1.1. Atrial Fibrillation

Atrial fibrillation (AF) is a disease characterized by a typical alteration of the cardiac rhythm; this pathology strikes 33 million people all over the world. Subjects affected by AF have a risk of cerebral ischemia 3–5 times higher than the average [129]. The prevalence of AF is 0.1% in adults aged <55 years and reaches 10% among those older than 80 years [130]. In wealthy countries, such as USA [130], statistics report the patients affected by this arrhythmia could double, and the strokes caused by cardioembolism derived from AF could triple in the next decades.

#### 9.1.2. Heart Failure

Almost 26 million people all around the world are affected by heart failure [131]. Even if in high-income countries this pathology always needs hospitalization more rarely [20], heart failure represents the most usual reason for admittance in hospitals. It constitutes almost 2% of the primary diagnoses at the moment of discharge. It seems that patients affected by heart failure are susceptible to forming cardiac thrombus when there are simultaneous states of local stasis or hypercoagulability and an untreated AF [132]. Consequently, this type of patient has a chance of stroke three times higher than the average [131].

#### 9.1.3. Patent Foramen Ovale

Almost 25% of the population has patent foramen ovale (PFO) [133]; this condition can represent a possible passage for a thrombus from the venous circulation to the arterial one causing a paradoxical embolism. Cryptogenic stroke, which represents approximately 20–40% of ischemic strokes, is described as a stroke that takes place without an identified cardioembolic or large vessel source and with a distribution that is not compatible with small vessel disease; most of these strokes are embolic. Patients who have experienced a cryptogenic stroke have an increased prevalence of PFO [134]; thus, paradoxical embolism via PFO is one likely cause of stroke in this population. On the other hand, some evidence suggests that PFO alone is not correlated with a raised chance of recurrent ischemia. Even if we may think that PFO could be a potent cause of cerebral ischemia, actually it is not, maybe excluding patients younger than 50 years.

#### 9.1.4. Myocardial Infarction

Another well-established possible cause of cerebral ischemia is the acute myocardial infarction (MI) [135]. We can observe that cardiac thrombi are often created in the ventricular portion that is dikinetic after previous cardiac ischemia. Additionally, the standard gold treatment for the acute cardiac ischemia, the percutaneous coronary intervention, has an intrinsic risk for ischemic stroke of ≈0.1% with the modern techniques [136]. Nowadays, better methods of reperfusion, the knowledge of the relevance of the antiplatelet therapy, and the optimized medical therapy for the secondary prevention have reduced the risk of cerebral ischemia compared to the past [137].

#### 9.1.5. Prosthetic Heart Valves

Around 2.5% of the general population is affected by moderate or severe valvular heart disease, and the percentage increases to 12% in people older than 75 [138]. As reported in a meta-analysis that examined the period between 1985 and 1992, the mechanical valve prosthesis causes a 4% annual hazard of stroke; the therapy with oral anticoagulants drastically reduces this percentage, specifically 1.3% for mitral prosthesis and 0.8% for aortic ones [139]. It seems that, compared to the mechanical prosthesis, the biological ones provoke a reduced risk of cerebral ischemia, this correlation is more reliable in the long term [140]. The complications caused by thromboembolism still represent a considerable risk for mortality and morbidity even if the general chance of stroke is decreased [141]. The traditional surgical aortic valve technique and the transcatheter one seem to have an equal chance of causing stroke [142].

#### 9.1.6. Infective Endocarditis

Around one in 10,000 persons in high-income settings experience infective endocarditis [143]. It is quite an unusual cause of cerebral ischemia. Still, the incidence of stroke in patients affected by endocarditis is relatively high; this pathology is complicated by cerebral ischemia in around 20% of the cases [143]. In the month following bacteriemia or infective endocarditis, the risk of cerebral ischemia is more than 20 times higher compared to the general population; this fact has been reported by various research [144].

#### 9.1.7. Other Causes

Other various pathological conditions that can rarely be a cause of cardioembolic stroke are represented by myxoma, mitral calcification, and papillary fibroelastoma. Each one causes less than 1% of strokes due to cardioembolism [145].

## 10. Diagnostic Criteria for Cardioembolic Stroke

It is necessary to verify that an embolus formed in the heart caused the cerebral ischemia to establish the diagnosis of cardioembolic stroke. Although the factors above lend a higher chance to provoke cardioembolism and consequent stroke, patients can be affected by other subtypes of stroke since they could have other risk factors. Therefore, it is not easy to differentiate this type of stroke from others. Thus, exact diagnosis requires the integration of clinical presentation, neuroimaging, vascular and cardiac evaluation. The typical characteristic of cardioembolic stroke is the sudden onset of neurological symptomatology that is more evident at the beginning.

On the other hand, a progressive neurological deficit is average of cerebral ischemia provoked by atherosclerosis of large arteries or occluded small vessels (lacunar stroke) [146]. In ischemic stroke caused by cardioembolism, the arteries involved are the ones furnishing the cerebral cortex, instead lacunar stroke concerns vessels of the subcortical area [147], making it possible to distinguish cardioembolic stroke by the presence of cortical symptoms, the reduction of the visual filed or aphasia for example. However, we cannot determine the origin of the cerebral ischemia just by the clinical presentation [147]. Researchers investigated radiological patterns of cerebral ischemia in patients affected by cardiac conditions with high chance to cause embolism and no other possible origins of stroke, so it was possible to establish typical neuroimaging findings of cardioembolic stroke. In particular, this subtype of stroke presents the involvement of cortical areas in most of the cases [148].

Furthermore, in 50% of the bouts of ischemic stroke caused by cardioembolism, various cerebral arterial territories are involved at the same time (i.e., the two internal cerebral arteries or just one of the internal cerebral artery and the basilar artery simultaneously) [148]. This fact makes distinct embolism with a cardiac source and the one caused by ulcerated atherosclerotic plaques. During the first stages, we can often observe an interruption of a vessel with no narrowing in the upstream arteries caused by atherosclerosis thanks to neurovascular imaging by magnetic resonance or computed tomographic [149]. To determine the diagnosis of cardioembolic stroke, the majority of strokologists exclude the possibility of carotid stenosis or intracranial plaques executing vascular imaging. It is necessary to perform other diagnostic investigations, such as a 12-lead ECG and transthoracic echocardiography, to identify the sources of cardiac thrombus.

## 11. Inflammation Background in Cardioembolic Stroke

Accumulating evidence proves that ischemic stroke starts an intricate process with genomic, molecular, and cellular modifications; phlogosis plays a crucial role in this mechanism, both in the CNS and in the periphery. At the same time, systemic inflammation seems to have a role in the pathological processes that cause cerebral cardioembolism [150] suggesting that CEI would present a higher inflammatory substrate than the other subtypes of ischemic stroke. Nevertheless, to date, only a few studies examined the inflammatory background of CEI. Tuttolomondo et al. [151,152] researched to observe if subjects affected by acute cerebral ischemia had variations of the number of selectins, cytokines, and adhesion molecules in relationship with the TOAST subtype. They also wanted to establish a possible association between the severity of the neurological symptomatology and the immunological background. They enrolled patients affected by acute cerebral ischemia and controls with no background of stroke. The stroke group was divided depending on the etiology according to the TOAST classification, in particular, LAAS, lacunar, CEI, and ODE. Patients affected by cerebral ischemia, compared to the control group, had an increased quantity of cytokines and adhesion molecules in plasma, such as E-selectin, P- selectin, V-CAM-1, ICAM-1, IL-1, IL-6, and TNF-α. In particular, the group of strokes classified as CEI presented relevantly elevated plasma concentrations TNF-α, IL-6, and IL-1 in comparison with the other strokes. Moreover, the CEI group displayed also slightly increased plasma levels of von Willebrand factor (vWF) compared to the rest of the stroke groups. Patients with CEI showed a significantly lower median Scandinavian Stroke Scale (SSS) score than patients with other subtypes of stroke. Based on these results, the authors concluded that CEI causes a more serious neurological deficit and consequently stroke with worse prognosis; this fact could be a result of the remarkable inflammatory process that takes place in the first phase of the disease.

Nakase et al. [153] researched to measure the quantity of the indicators of phlogosis during acute cerebral ischemia, to evaluate the possible difference between the subtypes and the correlation with the prognosis. They enrolled 105 subjects affected by the pathology within a day following the beginning of the symptomatology. They divided patients into different groups considering the diverse etiologies of stroke, in particular CEI, LAAS, lacunar, and the ones caused by arterial dissection and branch atheromatous disease. The division was made according to clinical records and the findings observed by magnetic resonance angiography (MRA) and MRI. The authors considered as markers of phlogosis the high-sensitive CRP (hsCRP), IL-6, and TNFα, which were measured at the admission and 28 days later. All the different groups showed similar serum concentrations of TNFα and IL-6. The CEI group just displayed relatively higher levels of hsCRP, which might represent a marker of myoendothelial injury in subjects affected by atrial fibrillation.

The results of these studies suggest that subjects affected by CEI are characterized by a relevant inflammatory milieu, considering plasma cytokines concentration, compared to other subtypes of cerebral ischemia. It is possible to determine some reasons for this evidence. The remarkable phlogosis that characterizes CEI strokes could be related to the more serious neurological symptomatology, generally due to the larger cerebral parenchyma involved, in comparison with the other varieties of stroke. Growing evidence supports the fact that there is a condition of neuroinflammation following an ischemic stroke that leads to an increased release of cytokines and chemokines that stimulate infiltration of leukocytes in the cerebral parenchyma. Therefore, extensive areas of cerebral tissue involved in the ischemic process, such as the ones observed in CEI strokes, could induce a higher neuroinflammatory response. The influence of stroke severity and infarct volume on the inflammatory response is supported by the study conducted by Tuttolomondo et al. [151]. In this study, a brain CT was routinely performed on patient admission and repeated at 4–7 days after stroke onset. Patients with LAAS subtype of acute ischaemic stroke, compared with subjects with cardio-embolic subtype had a lower mean brain infarct volume (11,894 vs. 12,273 mm^3^) and significantly lower median plasma values of TNF-α, IL-6, and IL-1β.

AF could provoke more serious cerebral ischemia and “longer” transient ischemic attacks (TIAs) than emboli from carotid disease, presumably because AF is responsible for the embolization of larger thrombi [154]. This correlation was analyzed by comparing ischemic cerebral accidents in patients with AF and those with a carotid disease in two significant trials: the proportion of hemispheric events to retinal events was 25:1 with AF compared with 2:1 with the carotid disease [154]. As a result, subjects affected by an ischemic stroke caused by AF seem to have a worse outcome (more disability, higher mortality) than those with no AF, even after adjustment for the advanced age of patients with AF-related stroke [155]. The “longer” TIAs typical in AF patients are more often characterized by abnormal magnetic resonance diffusion imaging and can be considered as strokes by the revised American Heart Association definition [156].

Furthermore, various research [157,158,159,160] reported a usual condition of phlogosis in patients affected by atrial fibrillation, hypothesizing that the inflammation could be a possible factor in the development and the evolution of this arrhythmia. Inflammation induces infiltration of the leukocytes into the intimae through the endothelium; adhesion molecules situated on the endothelial surface are involved in this process initiating the capture and the rolling of the leucocytes along the endothelium. Therefore, the amplified inflammatory background that characterizes ischemic stroke could be justified by a more relevant local neuroinflammatory response consequent to a more severe ischemic damage as well as by the presence of risk factors for cardioembolism such as atrial fibrillation, which is characterized by an intrinsic phlogosis. The result would be a more enhanced inflammatory milieu than other ischemic stroke subtypes. The neuroinflammatory mechanism characterizes all the subtypes of cerebral ischemia; however, it is also true that the different subtypes such as large artery atherosclerosis (LAAS) and lacunar infarct (LAC) do not have the same risk factors as the CEI (such as atrial fibrillation). This fact could explain the peculiar inflammatory environment that follows cardioembolism and the poor prognosis that characterizes this type of ischemic stroke.

In conclusion, cardioembolism causes ischemic brain injury that is induced by an intricate mechanism that involves genomic, molecular, and cellular levels resulting in a higher inflammatory environment than the other stroke subtypes. To date, data on neuroinflammation associated with cardioembolism are limited. Therefore, future studies are necessary to analyze accurately the inflammatory background of this stroke subtype and its underlying mechanisms to be able to develop more efficacious treatments.

## 12. Therapeutic Strategy for Cardioembolic Stroke

AF is a significant risk factor for the development of CEI. In such subjects, a cardiac embolus most commonly originating from the left atrial appendage is a common source of cerebral ischemia. All patients affected by acute ischemic stroke, including the ones with cardioembolism, should be evaluated to ascertain eligibility for reperfusion therapy with intravenous thrombolysis using alteplase (tPA) or mechanical thrombectomy; aspirin and other antithrombotic agents should be postponed until 24 h following treatment with intravenous tPA. Specific evidence on the efficacy of thrombolytic therapy in ischemic stroke is limited for patients with atrial fibrillation (AF), although such subjects account for 20–30% of those involved in clinical trials [161]. Otherwise, in the absence of contraindications such as severe systemic or intracranial bleeding, acute antithrombotic therapy may be justified in subjects affected by AF who suffer cerebral ischemia, both to reduce disability and the chance of early recurrent stroke, which is 3–5% during the first 14 days. While parenteral anticoagulation is usually disapproved for treating cerebral ischemia during the acute phase, using a direct oral anticoagulant (DOAC) or warfarin is recommended for secondary stroke prevention in subjects affected by atrial fibrillation or presenting other high-risk sources of cardiogenic embolism. Evidence from the pivotal clinical trials (RE-LY, ROCKET AF, ARISTOTLE, and ENGAGE AF-TIMI 48) [162] indicates that warfarin is not superior to DOACs in subjects affected by non-valvular atrial fibrillation for the prevention of systemic embolism or cerebral ischemia. DOACs were also associated with a relevant reduction in hemorrhagic stroke and intracranial hemorrhage compared to warfarin with a consequent decreased risk of stroke and mortality. Furthermore, subjects treated with DOACs suffered from less severe major bleedings than the ones treated with warfarin. The timing of oral anticoagulation initiation for such patients is mainly dependent on the size of the infarcted area and the presence of factors such as symptomatic hemorrhagic transformation and scarcely controlled hypertension [163]. Oral anticoagulation can begin immediately for subjects with a TIA due to atrial fibrillation, and a few hours after the appearance of the neurologic symptomatology (such as at the moment of hospital discharge or 24–48 h following stroke onset) for medically stable patients affected by a minor stroke (clinically defined by an NIHSS score ≤ 3) and no bleeding complications. For subjects with large infarctions (clinically defined by an NIHSS score > 15) or poorly controlled hypertension, withholding oral anticoagulation for 1–2 weeks is generally recommended. Finally, for cardioembolic stroke patients with symptomatic hemorrhagic transformation, it is recommended to delay antithrombotic treatment until the resolution of the bleeding and the clinical stabilization; then it is possible to switch to oral anticoagulation.

Besides, other strategies capable of reducing risk factors are needed in subjects affected by cardioembolic stroke. Some behavioral and lifestyle modifications may be useful for decreasing the risk of cerebral ischemia and cardiovascular disease. These include smoking cessation, limited alcohol consumption, weight control, regular aerobic physical activity, salt restriction, and a Mediterranean diet. Moreover, adequate therapy for blood pressure control is a relevant part of the management of subjects affected by AF with a previous stroke. Antihypertensive treatment, preferably including an angiotensin-converting enzyme inhibitor, decreases the risk of intracranial hemorrhage induced by warfarin and may lessen the rate of recurrent stroke. In a secondary analysis from the PROGRESS trial, among the subset of 476 patients affected by AF, therapy with perindopril resulted in a mean 7.3/3.4 mmHg reduction in blood pressure compared to placebo and a 34% decrease in the incidence of recurrent stroke (13.6% vs. 18.9%); nevertheless, this difference was not statistically relevant because of the limited number of recurrent events [164]. However, there was a significant 38% reduction in all major vascular events (one major vascular event prevented in every 11 patients treated for 5 years), establishing a strong rationale for blood pressure lowering. Another essential treatment is represented by statin therapy. Statin therapy decreases the chance of recurrent cerebral ischemia and cardiovascular events among subjects affected by a stroke of atherosclerotic origin. However, the efficacy of therapy with a statin has not been well explicitly established for subjects affected by CEI. However, a report of 6116 patients affected by cerebral ischemia showed that outpatient adherence to statin therapy was correlated with a decreased chance of recurrent ischemic stroke for patients with AF as well as those without AF, even after adjustment for the time in the therapeutic range of the international normalized ratio among patients with AF taking warfarin [165].

In conclusion, about 10% of AF patients with ischemic stroke or TIA have cervical carotid stenosis of 50% or greater diameter stenosis, slightly more than half of which are ipsilateral to the neurological symptoms. Based on estimates of attributable risk, ipsilateral stenosis of at least 70% is equally likely to be the cause of cerebral ischemia as cardiogenic embolism. Consequently, carotid endarterectomy or stenting appears to be rational for subjects affected by AF with high-grade ipsilateral stenosis, followed by chronic anticoagulation and antiplatelet therapy, although this approach is empiric, without proper supporting evidence.

## 13. Potential Treatment Strategies in Neuroinflammation after Ischemic Stroke

Accumulating evidence demonstrates that the process of phlogosis has a crucial function in the development of the ischemic damage playing a bivalent role; in fact, it promotes the loss due to ischemia and the healing of the parenchyma involved. Consequently, neuroinflammation has become a promising target for future therapies. The anti-inflammatory strategies have the aim of reducing phlogosis inhibiting its main elements such as microglia, T lymphocytes, and other agents with a proinflammatory action, for example, the cytokines (see Table 1).

One of the cytokines most involved in the development of the ischemic injury is IL-1. In mice lacking just one of the IL-1 isoforms (IL-1a and IL-1b), we did not observe a significant reduction of the damaged due to ischemia. On the other hand, mice that were deficient of both forms of the cytokine had a relevant decrease of the cerebral area affected by ischemia in comparison with the wild type ones (total amount: 70%; cortex: 87% reduction) [166]. Earlier research reported a significant reduction of the area involved in ischemia after MCAO or damage caused by excitotoxicity after the inoculation of a low dosage of recombinant human interleukin-1 receptor antagonist (rhIL-1ra) into the brain of rats. Specifically, Martin et al. [167] proved the beneficial result of the injection of rhIL-1ra in 7-day-old rats after the exposition of their cerebral tissue to a condition of ischemia and consequent hypoxia made by the ligation of a carotid artery. Another study [168] reported a significant reduction of neurons’ death after the administration of IL-1ra even if postponed until 3 h after the onset of a focal and not permanent cerebral ischemia in rats. Consequently, we can hypothesize that this antagonist might have a crucial function in possible treatments for stroke. Additionally, the neurological results in rats after reversible MCAO were better after the administration of antibodies anti-TNF-α [169], enhancing once again that this cytokine has a damaging action and the possible relevance of its antibodies in preserving the cerebral tissue from the harm caused by the reperfusion.

Previous research demonstrated the beneficial role of the administration of IL-10 in mice after permanent MCAO. Transgenic mice expressing IL-10 (IL-10T), compared to the wild type ones, had an excessive production of IL-10 by endothelial cells, microglia, and astrocytes. After 4 days from MCAO, it was possible to observe in IL-10T mice a 40% reduction of the area affected by ischemia, with a consequent significant reduction of the levels of caspase 3, compared to the wild type ones [170]. Insulin-like growth factor 1 (IGF-1), when given subcutaneously, caused a substantial reduction of the ischemic area and an improvement of sensibility and mobility in mice with normal levels of blood pressure [171].

Another potential therapeutic strategy is the modulation of the microglial response by modulating the expression of Toll-like receptors (TLRs). TLRs are part of the innate immune system able to stimulate a precise reaction after a systemic infection caused by bacteria. In microglia cells, TLRs can induce the expression of genes for cytokines with proinflammatory function [172]. Microglia expresses mainly TLR4 and TLR2, even more after stroke [173]. Furthermore, it was observed an increased presence of TLR4 on CD11b-positive microglia cells in the striatum involved in stroke [174]. Compared with wild-type mice, knockout (KO) ones for TLR4, but not for TLR3 or TLR9, showed the extension of cerebral tissue affected by the ischemia relevantly reduced 24 h after induced ischemia and the subsequent reperfusion [174]. Early research, studying the role of TLR2 in the cerebral tissue affected by ischemia, reported in TLR2-KO mice a reduced activation of macrophage/microglia and consequently their ability to increase compared to the controls. Moreover, they reported a decreased quantity of levels of monocyte chemotactic protein-1 (MCP-1), which caused the reduction of cells capable of expressing CD45 high/CD11b^+^ [175].

Furthermore, various studies, demonstrating the crucial function played by T cells in ischemic stroke, proposed this cytotype as a possible target for new treatments: in fact, they observed that the deficiency of the subsets of T cells in animal models resulted in a smaller area of cerebral parenchyma injured by ischemia [29,176]. A further study [177] described the interference of the cerebral invasion by the inhibition of leukocyte very late antigen-4 and endothelial vascular cell adhesion molecule-1 as a promising strategy of treatment against the neuroinflammation caused by ischemia in murine models. The inactivation of the first antigen by monoclonal antibodies provoked the reduction of the leukocytes’ number in the ischemic tissue and the releasing of toxic cytokines with no risen susceptibility to bacterial infection during cerebral ischemia of moderate entity. Even the blockade of the second antigen, the vascular one, by the injection of interfering RNA provoked similar results. Furthermore, very late antigen-4 inhibition significantly reduced the expression of vascular cell adhesion molecule-1 after cerebral ischemia; consequently, we can hypothesize the presence of cross signaling between endothelial cells of the cerebral vessels and the leucocytes activated by inflammation.

Multiple studies investigated the possible therapeutic role of hypoxia-inducible factor-1, known as HIF-1. This is a transcription agent formed by two subunits (α and β) whose production is stimulated by hypoxia and has been found in mice cerebral tissue affected by an ischemic stroke. It has various abilities such as promoting the secretion of erythropoietin (EPO) and vascular endothelial growth factor (VEGF) and the survival of the cerebral cells through the repression of p53, caspases, and the cytochrome c [178]. Guo et al. reported that the stimulation of the subunit HIF-1α resulted in decreased cell death in cerebral tissue affected by ischemia. On the other hand, its inhibition by using small interfering RNA (siRNA) provoked the overexpression of reactive oxygen species and consequently, an increased cerebral injury [179]. On the contrary Chen et al. observed that the suppression of this subunit was associated with a better outcome because it provoked the inhibition of BCL2/adenovirus E1B 19 kDa protein-interacting protein 3 (BNIP3) which is responsible for mitochondrial dysfunction [180]. These differences might be justified by the different timing of the ischemic process; in fact, Baranova et al. reported the beneficial effect of HIF-1α after a 30-min MCAO [181], Helton et al. instead observed opposite results after 75-min bilateral occlusion of common carotids [182]. Possible future therapeutic strategies could be based on the stimulation of this transcription factor in the early stage of ischemia and a successive suppression during the next phases.

Another possible target for future therapies is Heme-oxygenase-1 (HO-1); the production of this enzyme is promoted by the transcription agent “nuclear factor erythroid 2-related factor 2” (Nrf2) after an oxidative injury. HO-1 can degrade heme with a consequent release of carbon monoxide (CO), biliverdin-IXa and ferrous iron (Fe^2+^) [183]. These molecules seem to play an anti-inflammatory and anti-apoptotic role [184,185,186,187,188]. Chao et al. reported a reduction of the cerebral tissue affected by ischemia and improvement of the neurological symptomatology in MCAO rats treated with viral carriers expressing HO-1 [189]. Panahian et al. observed a similar result in transgenic mice expressing HO-1 [190]. On the other hand, Shah et al. showed that HO-1 knock-out mice had increased extension of the cerebral area involved in ischemia than the wild-type control group [191]. Therefore, we can deduce that the stimulation of the pathway Nrf2-HO-1 could represent a possible target for future treatments.

At the moment, apart from mechanical thrombectomy, the only pharmacological therapy approved for acute ischemic stroke is the recombinant tissue plasminogen activator (rtPA). This treatment has a short timeframe in which the patient could receive it (3–4.5 h from the onset of the neurological symptomatology) and has several contraindications due to its intrinsic high bleeding risk [192]. Due to these properties, just the 3–5% of patients affected by acute ischemic stroke admitted in hospitals are eligible for this therapy [193]. Furthermore, this drug does not affect at all the inflammatory mechanism which takes place in the cerebral tissue after the ischemic injury and has a crucial role in influencing the prognosis. Consequently, the development of alternative therapeutic strategies, even combined with rtPA, appears to be decisive to improve the outcome of patients affected by an acute ischemic stroke.

## 14. Discussion

The pathophysiology of stroke is a complex process, and always more findings propose that cerebral ischemia has a background characterized by phlogosis. According to this point of view, the inflammatory reaction that occurs after the neuronal injury, also called neuroinflammation, plays a significant role. Neuroinflammation in ischemic stroke is exceptionally elaborate, with multiphasic proinflammatory responses. Indeed, in the setting of ischemic neuronal damage, the release of cytokines, chemokines, and ROS promotes the development of phlogosis in the cerebral parenchyma. The reduced perfusion and the consequent hypoxia encourage the production of ROS by immune cells in the brain provoking oxidative stress and the activation of the cells of the endothelium. In the ischemic context, ROS directly damage the vascular structure and start the process of inflammation correlated with an immediate immune response. Following the ischemia, glial cells, such as astrocytes and microglia, endothelial cells, and leucocytes, release various agents with a proinflammatory action, such as chemokines, cytokines, and other enzymes. Thus, inflammation and the immune system injure the cerebral parenchyma by a multi-step process that starts when monocytes and leucocytes reach the brain, then we can observe the triggering of resident cells, such as the endothelial ones, astrocytes, and microglia, and the consequent release of various proinflammatory cytokines. These cooperate in developing cerebral damage due to ischemia; this fact has been shown both in animal models and in patients affected by an ischemic stroke. We can observe the cells of the immune system involved in this process in the injured areas not simultaneously. Furthermore, various evidence shows that the cells involved in phlogosis have a divalent function (helpful and harmful); in fact, the same pathway inactivated at different times could exacerbate or reduce the damage of the cerebral parenchyma. Therefore, possible future therapies should consider the timing of the ischemia. Moreover, multiple genetic factors involved in phlogosis could modulate the process of inflammation. Many genes are capable of influencing the nature of the cerebral ischemia and the extension of the area affected, modifying the prognosis. To date, neuroinflammation represents a complex event, regulated by several factors, which plays a crucial role not only in the pathogenesis of the ischemic damage but also in determining its evolution; consequently, inflammation during cerebral ischemia could be a promising target in the development of new therapeutic strategies.

Still, various studies report that each subtype of cerebral ischemia has a different pattern of inflammation that takes place in the brain. Among these, in CEI, it is possible to observe a more neurological severe symptomatology, assessed with SSS, and a more significant inflammation during the first stage of ischemia compared to the other subtypes. This evidence, concerning an exalted inflammatory milieu (in terms of plasma cytokines levels) in patients with CEI, highlights the importance of phlogosis in the development of cerebral damage in cardioembolic stroke. Various findings report that in patients affected by AF, there is a condition of inflammation that may be a possible cause of the development of this arrhythmia. Phlogosis induces the trans-endothelial migration of leukocytes into the intimae after their recruitment and adhesion mediated by specific molecules that make the rolling along the endothelium possible. On the other hand, ischemic brain damage of large cerebral areas, such as the ones produced by cardioembolic sources, could generate a higher inflammatory response. Hence, a better characterization of stroke pathophysiology and inflammatory background of each subtype, in particular of CEI, with time-defined anti-inflammatory treatment could be a fundamental step in developing better therapeutic approaches.

## Figures and Tables

**Table 1:** Experimental studies targeting neuroinflammation in stroke.

Target	Subjects	Result	Reference
Interleukin (IL)-1	IL-1a and IL-1b deficient mice	Subjects showed a reduction of the ischemic area compared to wild-type mice	H. Boutin, R.A. LeFeuvre, R. Horai, M. Asano, Y. Iwakura, and N.J. Rothwell, “Role of IL-1α and IL-1β in ischemic brain damage,” The Journal of Neuroscience, vol. 21, no. 15, pp. 5528–5534, 2001.
IL-1	Mice treated with rhIL-1ra after the ligation of a carotid artery	Subjects showed a reduction of neurological deficit	Martin D, Chinookoswong N, Miller G. The interleukin-1 receptor antagonist (rhIL-1ra) protects against cerebral infarction in a rat model of hypoxia-ischemia. Exp Neurol 1994; 130(2): 362–367.
Tumor necrosis factor (TNF)-α	Mice treated with antibodies anti-TNF-α after reversible temporary occlusion of the middle cerebral artery (MCAO)	Subjects showed a better neurological outcome	Lavine SD, Hofman FM, Zlokovic BV. Circulating antibody against tumor necrosis factor-alpha protects rat brain from reperfusion injury. J Cereb Blood Flow Metab 1998; 18(1): 52–58.
IL-10	IL-1T mice after permanent MCAO	Subjects showed a 40% reduction of the area involved in ischemia	F. de Bilbao, D. Arsenijevic, T. Moll et al., “In vivo overexpression of interleukin-10 increases resistance to focal brain ischemia in mice,” Journal of Neurochemistry, vol. 110, no. 1, pp. 12–22, 2009
Insulin-like growth factor 1 (IGF-1)	Mice treated with IGF-1 subcutaneously after permanent MCAO	Subjects showed a reduction of the ischemic area with an improvement of sensibility and mobility	D. de Geyter, W. Stoop, S. Sarre, J. de Keyser, and R. Kooijman, “Neuroprotective efficacy of subcutaneous insulin-like growth factor-I administration in normotensive and hypertensive rats with an ischemic stroke,” Neuroscience, vol. 250, pp. 253–262, 2013.
TLR4	Knock out mice for TLR4 24 h after induced cerebral ischemia and successive reperfusion	Subjects showed a reduction of the ischemic area compared to wild-type mice	K. Hyakkoku, J. Hamanaka, K. Tsuruma et al., “Toll-like receptor 4 (TLR4), but not TLR3 or TLR9, knock-out mice have neuroprotective effects against focal cerebral ischemia,” Neuroscience, vol. 171, no. 1, pp. 258–267, 2010.
T cells	Mice deficient in T cell subsets	Subjects showed a smaller ischemic area compared to wild-type mice	Hurn PD, Subramanian S, Parker SM, et al. T- and B- cell-deficient mice with experimental stroke have reduced lesion size and inflammation. J Cereb Blood Flow Metab 2007; 27: 1798–1805.
Leukocyte very late antigen-4 and endothelial vascular cell adhesion molecule-1	Mice affected by cerebral ischemia	The inhibition of these molecules resulted in a reduction of the leukocytes’ recruitment in ischemic parenchyma with consequent decreased neuronal damage	Liesz A1, Zhou W, Mracskó É, Karcher S, Bauer H, Schwarting S, Sun L, Bruder D, Stegemann S, Cerwenka A, Sommer C, Dalpke AH, Veltkamp R. Inhibition of lymphocyte trafficking shields the brain against deleterious neuroinflammation after stroke. Brain. 2011 Mar;134(Pt 3):704–20. DOI: 10.1093/brain/awr008.
Hypoxia-inducible factor (HIF)-1α	Ischemic cerebral tissue in vitro	The stimulation of HIF-1α resulted in a reduced neuronal death, on the other hand, its suppression by small interfering RNA (siRNA) was associated with increased production of reactive oxygen species (ROS)	Guo S, Miyake M, Liu KJ, Shi H. Specific inhibition of hypoxia-inducible factor exaggerates cell injury induced by in vitro ischemia through deteriorating cellular redox environment. J Neurochem 2009; 5:1309–1321.
HIF-1α	Mice after induced MCAO	The inhibition of HIF-1α was associated with a better neurological outcome by the suppression of BNIP3 (BCL2/adenovirus E1B 19 kDa protein-interacting protein 3) which is responsible for mitochondrial dysfunction	Chen C, Hu Q, Yan J, Lei J, Qin L, Shi X, Luan L, Yang L, Wang K, Han J, Nanda A, Zhou C., Multiple effects of 2ME2 and D609 on the cortical expression of HIF-1α and apoptotic genes in a middle cerebral artery occlusion-induced focal ischemia rat model. J Neurochem. 2007
HIF-1α	Mice after 30-min MCAO	The inhibition of HIF-1α was associated with a worse neurological outcome	Baranova O, Miranda LF, Pichiule P, Dragatsis I, Johnson RS, Chavez JC. Neuron-specific inactivation of the hypoxia-inducible factor 1α increases brain injury in a mouse model of transient focal cerebral ischemia. J Neurosci 2007; 23:6320–6332.
HIF-1α	Mice after 75-min bilateral occlusion of carotid arteries	The stimulation of HIF-1α was associated with a worse neurological outcome	Helton R, Cui J, Scheel JR, Ellison JA, Ames C, Gibson C, Blouw B, Ouyang L, Dragatsis I, Zeitlin S, Johnson RS, Lipton SA, Barlow C. Brain-specific knock-out of hypoxia-inducible factor-1α reduces rather than increases hypoxic-ischemic damage. J Neurosci 2005;16: 4099–4107.
Heme oxygenase (HO)-1	Rats treated with viral carries expressing HO-1 after MCAO	Subjects showed a reduction of the ischemic area and an improvement of the neurological symptomatology	Chao XD, Ma YH, Luo P, et al. Up-regulation of heme oxygenase-1 attenuates brain damage after cerebral ischemia via simultaneous inhibition of superoxide production and preservation of NO bioavailability. Exp Neurol 2013; 239: 163–9.
HO-1	Transgenic mice expressing HO-1 after permanent MCAO	Subjects showed a reduction of the ischemic area and an improvement of the neurological symptomatology	Panahian N, Yoshiura M, Maines MD. Overexpression of heme oxygenase-1 is neuroprotective in a model of permanent middle cerebral artery occlusion in transgenic mice. J Neurochem 1999; 72: 1187–203.
HO-1	HO-1 knockout mice affected by cerebral ischemia	Subjects showed an increased ischemic area compared to wild-type mice	Shah ZA, Nada SE, Dore S. Heme oxygenase 1, beneficial role in permanent ischemic stroke and in Gingko biloba (EGb 761) neuroprotection. Neuroscience 2011; 180: 248–55.
